# Comparing treatment of proximal phalangeal fractures with intramedullary screws versus plating

**DOI:** 10.1007/s00402-022-04516-z

**Published:** 2022-08-22

**Authors:** Kaspars Silins, Tutku Turkmen, Esther Vögelin, Luzian C. P. Haug

**Affiliations:** 1 Department of Hand Surgery, Inselspital University Hospital of Bern, Schänzlistrasse 33, 3010 Bern, Switzerland; 2 Faculty of Computer Science and Information Technology, Riga Technical University, Ķīpsalas iela 6A, Kurzemes rajons, Riga, LV-1048 Latvia

**Keywords:** Phalangeal fracture, Proximal phalanx, Intramedullary screw osteosynthesis, Plate

## Abstract

**Purpose:**

Phalangeal fractures are the most common injuries in humans and account for approximately 10% of all fractures. With plate fixation, anatomic reduction is achievable in most cases, but extension lag is seen in up to 67%. Intramedullary headless screw offers treatment of unstable proximal phalangeal fractures using a minimally invasive procedure with very few complications. One of the major disadvantages of this technique is the transarticular screw position, damaging the articular surface and thus preventing very proximal fractures from being treated with a distally inserted screw. In this study, we present a modified approach to the fixation of the proximal phalangeal fractures and compare outcomes with plate osteosynthesis.

**Materials and methods:**

Twenty-nine patients with 31 comparable fractures of the proximal phalanx were treated either with a plate (14) or with minimal invasive cannulated compression screw (17). Pain, strength, range of motion (ROM), work disability and QuickDASH score were assessed.

**Results:**

TAM was significantly better in the screw group. The extension lag was worse in the plate group. Plate removal had to be performed in 13 of 14 the cases, while the screw had to be removed in only 3 cases. The average duration of work disability was 9.9 weeks in the plate group, compared to 5.6 weeks in the screw group.

**Conclusion:**

Minimally invasive screw osteosynthesis not only has the advantage of significantly shorter work disabilities, but also shows remarkably improved postoperative range of motion. In contrast to plate osteosynthesis, removal of the screw is only necessary in exceptional cases. With the antegrade screws position, even difficult fractures close to the base can be treated without destroying any articular surface. In proximal phalanx fractures with both options of plate or single-screw osteosynthesis, we recommend minimal invasive cannulated screw osteosynthesis.

## Introduction

Phalangeal fractures are one of the most common injuries in the entire skeletal system and accounts for approximately 10% of all fractures [1]. Moreover, proximal phalangeal fractures are among those that most commonly affect the hand [2].

With plate fixation, an almost anatomic reduction is achievable in most cases, but an extension lag is seen in up to 67% of operated patients [3].

Intramedullary headless screw offers treatment of unstable proximal phalangeal fractures using a minimally invasive procedure. A few recent studies show good outcomes after this procedure with very few complications [4–7]. One of the major disadvantages of this technique is the transarticular screw position, which leaves a defect of the articular surface. This problem was studied by Borbas et al. in a cadaver study and they showed that the size of this defect can reach 8.5% of the articulating surface of the proximal phalanx [8]. In addition, transarticular screw fixation is not possible for fractures near the base of the phalanx and for more proximal fractures.

In this study, we present a modified approach to the fixation of the proximal phalangeal fractures introducing the intramedullary headless screw antegrade from the radial or ulnar base of the proximal phalanx. We compare the outcome with plate-fixation osteosynthesis in proximal phalangeal fractures.

## Materials and methods

Between February 2017 and March 2020, we selected 32 patients with 34 fractures of the proximal phalanx that were admitted and treated at our institution. Twenty were treated with a cannulated screw system (CSS) and 14 underwent a plate osteosynthesis. They were divided into two groups accordingly. We selected comparable fractures that were instable (mostly shaft and oblique fractures) and that could be treated with both methods regardless of mechanism of injury. The method was chosen by the respective surgeon. Exclusion criteria were fractures of the basic phalanx of thumb, pathological fractures, extensive soft tissue damage including amputation as well as patients not willing to take part in this study.

Twenty-one patients were male and 11 were female with the mean age of 46 (16–82) years. Eleven fractures affected the dominant hand. 25 fractures were closed, 9 were open. In the screw group, 4 of 17 fractures were open. Five of them were multifragmentary (two open and multifragmentary).

In the plate group, 3 of 14 fractures were open. Six of them were multifragmentary (two open and multifragmentary). Outpatient surgery was performed on 22 patients and inpatient surgery on 10 patients.

**Fig. 1:**
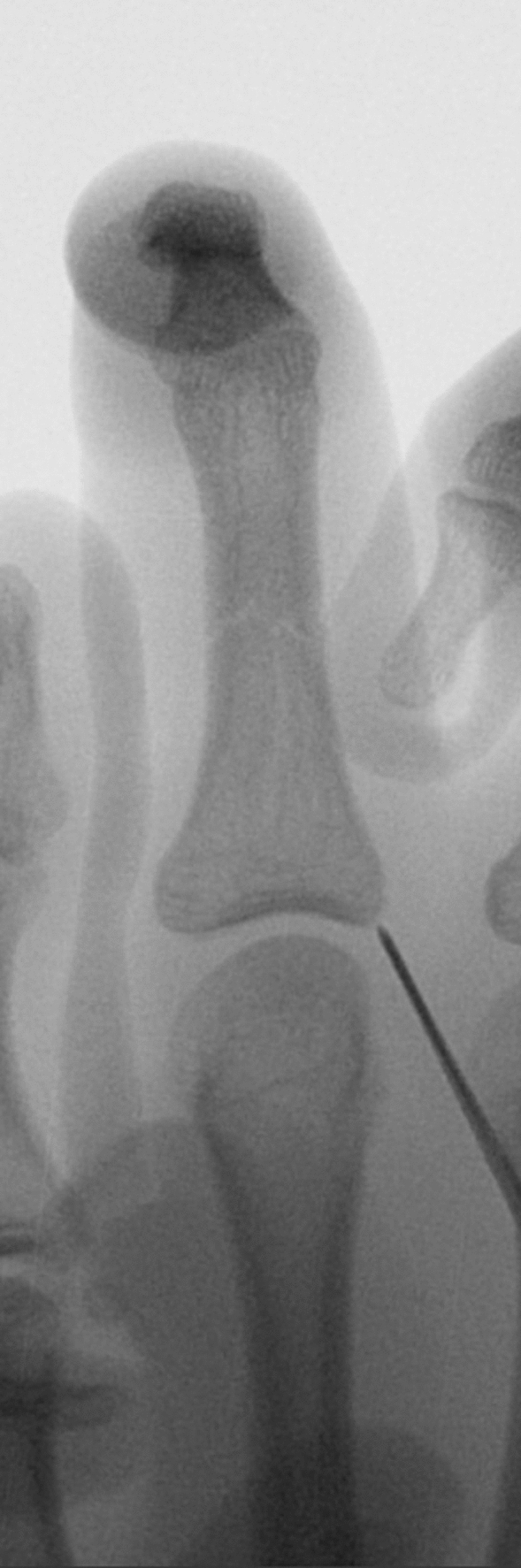
Entry point of the *K*-wire at the base of the proximal phalanx

In the screw group, we used a 1.7–3.0 mm self-tapping cannulated screw (Stryker Autofix/SpeedTip CSS Screw). All the screws were introduced in an antegrade manner. Depending on the fracture anatomy, we chose the entry point radial or ulnar at the base of the phalanx. The closed reduction of the fracture was done under fluoroscopy by traction and maximal flexion of the finger. *K*-wire was introduced percutaneously. The entry point shall be chosen either ulnar or radial (depending on the fracture anatomy) at the base of the proximal phalanx without injuring the metacarpophalangeal joint (see Fig. 1). After choosing the proper length, the cannulated screw was screwed in under a fluoroscopic view. After achieving good compression and obtaining radiographs, the *k*-wire was removed and the skin was sutured.

An intrinsic plus cast was applied after the surgery for the first 2 weeks. The patients were free to move the operated finger out of the cast without weight bearing. After 2 weeks, a shorter intrinsic plus cast was adjusted to be used at night for another 4 weeks. After 6 weeks, patients were allowed to slowly start strengthening the finger and the cast was no longer necessary.

The plate group received an open reduction and internal fixation of the fracture using a dorsal plate (Compact Hand VA). The postoperative management was identical to the screw group.

A postoperative follow-up consisted of a clinical and radiological evaluation after 6 and 12 weeks. Total active motion (TAM), grip and pinch strength were measured. Pain was evaluated using visual analogue scale (VAS) in resting position and under load.

The coronal and dorso-volar angles of the fracture were measured pre- and postoperatively with X-ray.

**Fig. 2:**
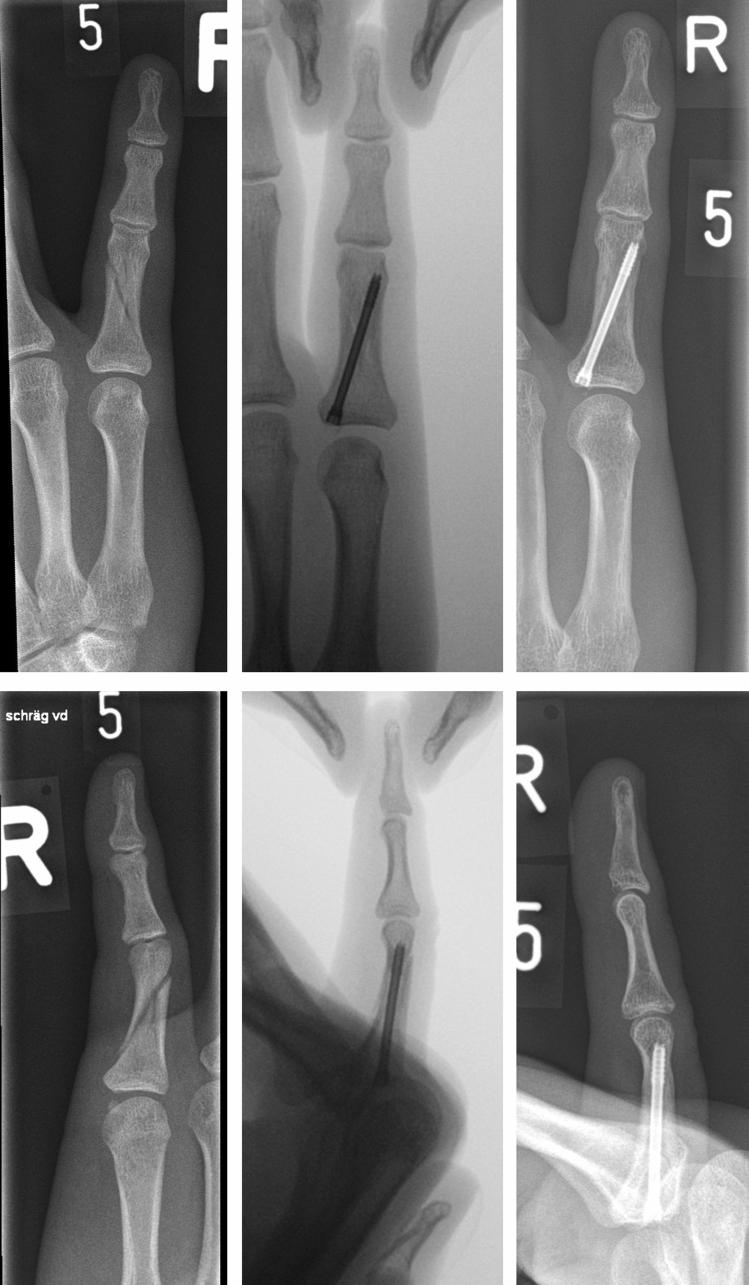

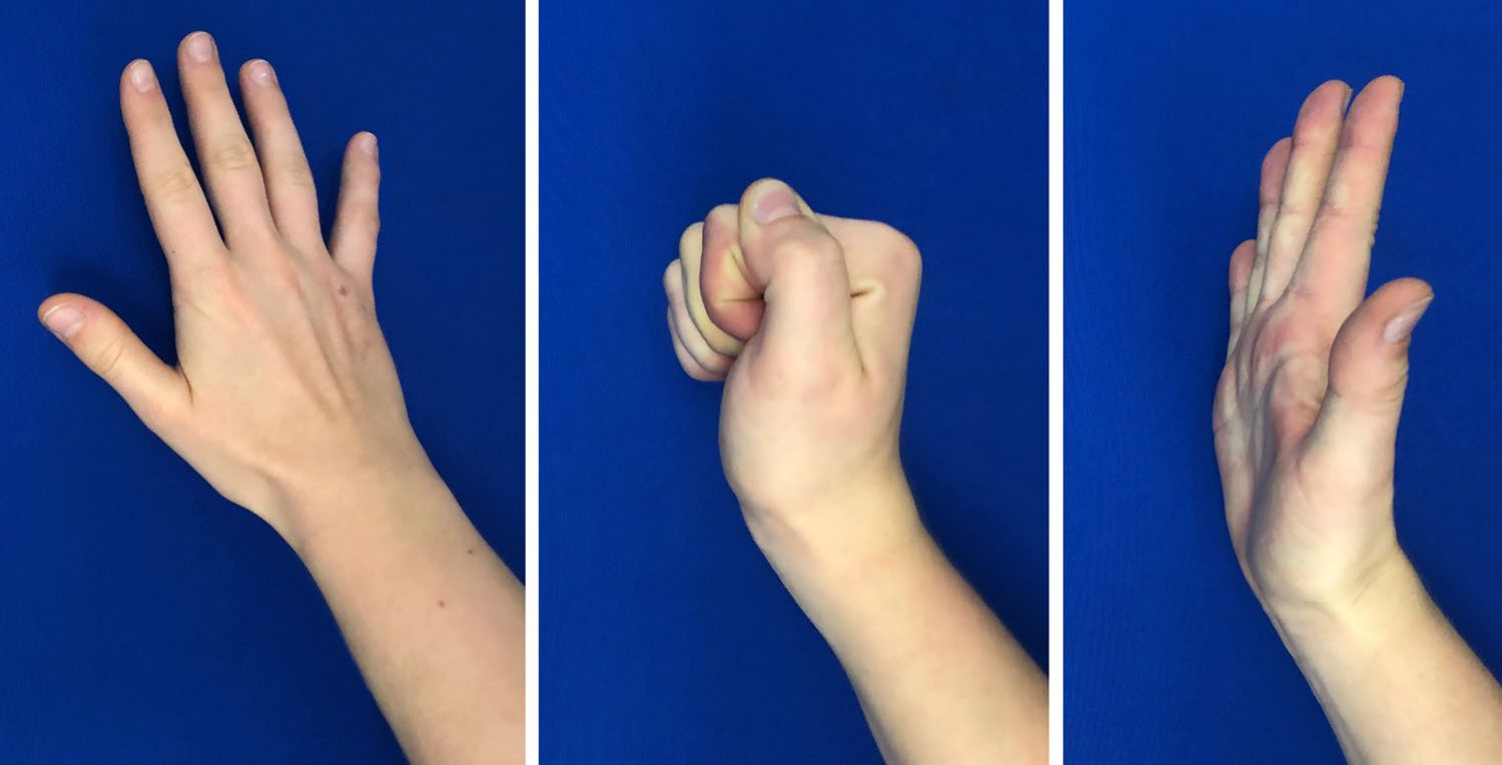
**a** First column shows the fracture. Second column shows intraoperative osteosynthesis of the fracture. Third column is the result after 6 weeks. **b** Clinical results 6 weeks after the operative treatment

An example of a treated proximal phalangeal fracture is seen in (Fig. 2a) and its clinical result in (Fig. 2b).

The QuickDASH questionnaire was completed at the last follow-up. Sick leave, surgery time and complications were registered. Informed consent was signed by all the patients.

For statistical analysis, we evaluated both groups comparing TAM, strength, pain, X-ray angles before and after the surgery, time to return to work as well as surgery time. Because of the small sample groups, the Mann–Whitney *U* test was used. The null hypothesis was defined as there being no difference between the two groups. The level of significance was set at *p* < 0.05.

Ethical approval for this study was given by the local ethics committee.

## Results

A total of 29 patients with 31 fractures were recruited for the study. Sixteen were in the screw group with 17 fractures, while 13 patients were in the plate group with 14 fractures. The average patient follow-up in the screw group was 9 months (range 5–16 months). The average follow-up in the plate group took place after an average of 9 months (range 4–12 months) after plate removal. The patients’ characteristics are summarized in Table 1.

**Table 1:** Patient’s demographics

	Group 1 (screw)	Group 2 (plate)
Patients, *n*	16	13
Fractures, *n*	17	14
Age, years (mean)	45 (16–82)	52 (30–71)
Gender, *n* (%)		
Male	12 (75%)	9 (69%)
Female	4 (25%)	4 (31%)
Work		
Physical work	8	5
Office work	5	4
Student	2	1
Retired	1	3
Dominant affected hand, *n* (%)		
Yes	8 (50%)	3 (23%)
No	8 (50%)	10 (77%)
Involved fingers		
Index	5	3
Middle	3	0
Middle	3	3
Little	5	7

The total active motion (TAM) was compared between both groups before and after removing the plate. Prior to the plate removal, the screw group showed a TAM of 246° while the plate group showed 205° with a difference of 41° between the two groups (*z* − 2.26, CI 95%, *p* = 0.02). After removing the plate the TAM in the plate group was 227° with a difference of 19° (*z* − 1.00, CI 95%, *p* = 0.32).

The mean flexion value of metacarpophalangeal (MCP), proximal interphalangeal (PIP) and distal interphalangeal (DIP) joint in the screw group was 87, 95 and 72° respectively. The plate group showed a mean flexion value of 85° (MCP), 79° (PIP) and 62° (DIP) before removing the plate. The difference value between both groups of PIP joint showed 16° which was statistically significant (*z *− 2.86, CI 95%, *p* < 0.01). After plate removal, mean values of 88° (MCP), 90° (PIP) and 77° (DIP) were recorded.

The mean extension lag in the screw group was measured at 1.2° in MCP joint, 5.9° in PIP and 0.3° in DIP joint, whereas the measurements after the plate osteosynthesis in the plate group 0, 18.9 and 2.5°, respectively. After removing the plate, the extension lag was measured at 2.1, 17.9 and 7.5°, respectively. The extension lag in PIP joint between the two groups before and after removing the plate was statistically significant (− 2.52, CI 95%, *p* = 0.01/*z* − 2.15, CI 95%, *p* = 0.03).

The screw group showed a mean grip value of 33.2 kg and pinch value of 9.4 kg. In the plate group, values of 33.1 kg and 9 kg were recorded, respectively (*z* − 0.02, CI 95%, *p* = 0.99/*z* − 0.74, CI 95% *p* = 0.46).

A visual analogue scale was used to evaluate pain. Both groups showed no pain in resting position (first group 0.06 versus 0.00 (*z *− 0.25, CI 95%, *p* = 0.79) and minimal pain under load (0.52 versus 0.92, respectively). There was no statistical significance between both the groups (*z* − 0.02, CI 95%, *p* = 0.98).

The postoperative angles in plain radiographs were measured. In the posterior–anterior (PA) view, the mean angle-deviation (radial or ulnar) in the screw group was 2.2° (0–13°) and in the plate group 1.6 (0–5°). These values showed no statistically significant difference (*z* − 0.02, CI 95%, *p* = 0.99). In the lateral view, the mean values of dorsal angulation were 4.4° (0–35°) and 2.2° (0–5°), respectively, which showed no statistical significance (*z *− 0.10, CI 95%, *p* = 0.92).

The QuickDASH score was filled out by both groups and the assessment showed mean % values of 3.2 in screw group versus 2.7 in the plate group with no statistical significance between these groups (*z* − 0.41, CI 95%, *p* = 0.68).

The screw group required a sick leave of 5.6 weeks (range 0–16 weeks) compared to 9.9 (2–22) weeks for the plate group including the time after the removal of the plate (*z* − 1.96, CI 95%, *p* = 0.05).

The average duration of the operation in the screw group was 36 min (18–64 min). The plate group showed an average duration of 71 min (47–98 min). There was a statistical significance between the groups (*z *− 3.75, CI 95%, *p* < 0.01).

**Fig. 3:**
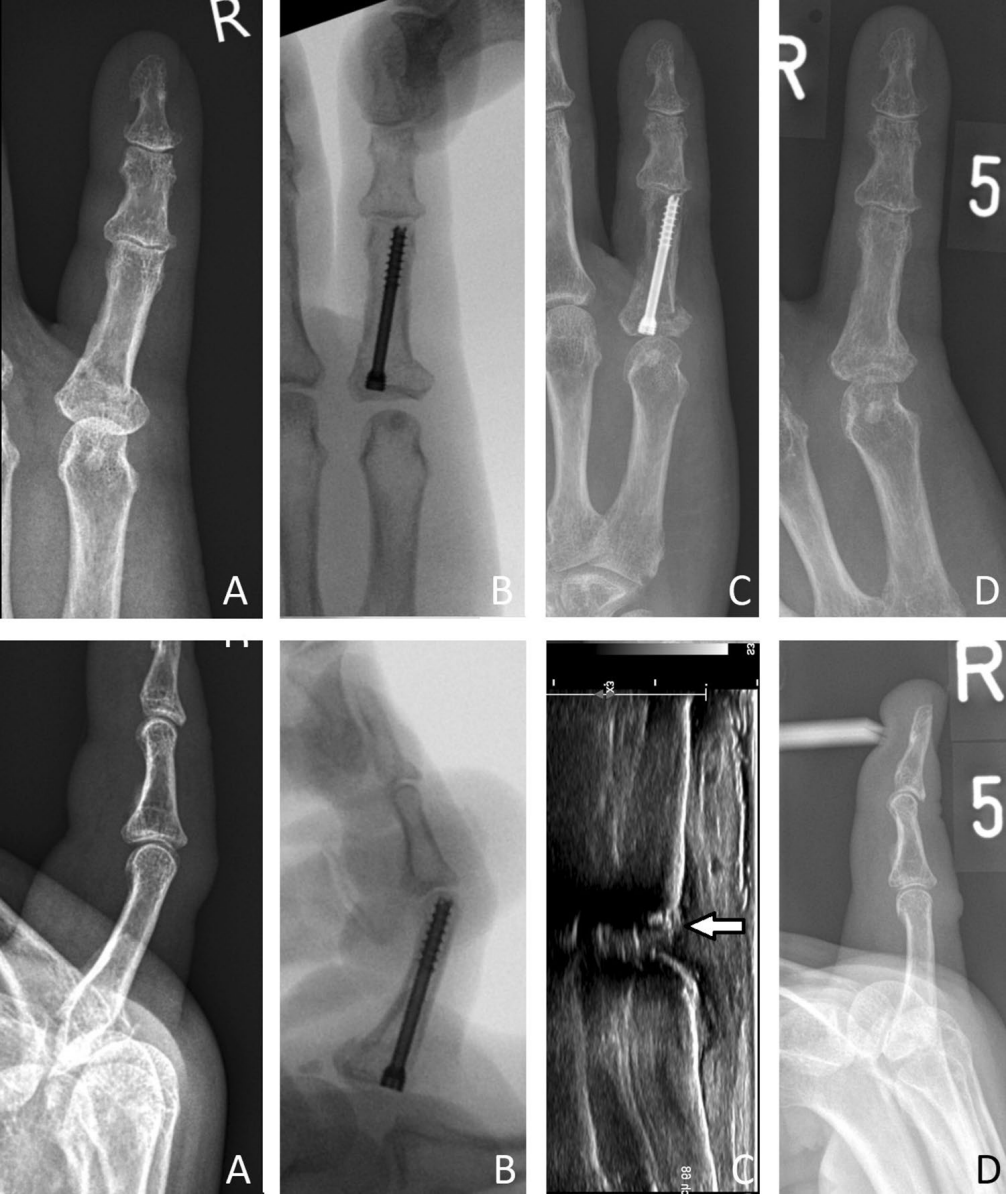
**A** First X-ray, **B** intraoperative radiographs, **C** 6 weeks postoperative. White arrow shows protruding screw in sonographic view, **D** after screw removal and healed fracture

In the screw group, 14 of 17 fractures healed without complications. In three cases (17.6%), the screw had to be removed. One patient suffered a dislocation of the screw in the metacarpophalangeal (MCP) joint which was associated with pain. An example is demonstrated in Fig. 3.

Another two patients suffered from a disturbing sensation due to the presence of the screw. In all three cases, the screw was removed through a stab incision.

In the plate group, the plate had to be removed in 13 of 14 cases (93%). In eight cases, an extension lag was present. In two cases, the screw was in contact with a tendon. In three cases, the plate removal was planned during routine follow-up. There were no infections documented in either group.

All the results are summarized in Table 2.

**Table 2:** Summary of results

	Group 1 (screw)	Group 2 (plate)	*p*-value
Patients	*N* = 16	*N* = 13	
Fractures, *n*	17	14	
Open fractures, *n*	4	3	
Multifragmentary, *n*	5	6	
Open and multifrg, *n*	2	2	
Follow-up (months)			
Mean (SD)	8.7 (2.6)	9.2 (2.6)	0.18
Type of fracture *n* (%)			
Close	13 (76)	11 (77)	–
Open	4 (24)	3 (23)	–
TAM mean° (SD)			
Injured finger	246 (24.2)	205 (55.8)	0.02
After plate removal	–	227 (45.2)	0.32
Uninjured finger	267 (18.3)	252 (22.8)	0.39
Flexion mean° (SD)			
MCP	87 (5.3)	85 (9.8)	0.46
PIP	95 (7.1)	79 (17.6)	< 0.01
DIP	72 (11.9)	62 (24.5)	0.35
Extension lag			
MCP	1.2 (3.3)	0 (0)	0.58
PIP	5.9 (8.9)	18.9 (14.8)	0.01
DIP	0.3 (1.2)	2.5 (5.8)	0.44
Strength kg (SD)			
Grip Jamar	33.2 (11.4)	33.1 (11.5)	0.99
Pinch	9.4 (1.9)	9(2.29)	0.46
Pain VAS			
At rest	0.05 (0.24)	0 (0)	0.79
At load	0.05 (0.24)	0.09 (2.29)	0.98
QuickDASH score			
Mean points (SD)	3.2 (6.5)	2.7 (4.9)	0.68
Radiologic outcome			
Coronal deviation	2.2 (3.1)	1.6 (1.6)	0.99
Dorsopalmar dev	4.4 (7.9)	2.2 (1.4)	0.92
Implant removal			
* n* (%)	3 (15.7)	13 (93)	< 0.01
Lack of work			
Weeks (SD)	5.6 (5.3)	9.9 (6.4)	0.05
Operation time			
Mean min (SD)	36 (14)	71 (18)	< 0.01

*TAM* total active motion, *SD* standard deviation, *VAS* visual analogue score

## Discussion

The goal of the fracture management is to allow the healing to take place in an eligible alignment without disturbing the gliding motion of the tendons. Lögters et al. 2018 published a management algorithm for proximal phalangeal fractures and concluded that an intramedullary screw fixation previously described shows no benefit compared to traditional plating [9]. Our study demonstrates a significantly better outcome in range of motion using the intramedullary screw osteosynthesis compared to plate osteosynthesis.

Another advantage of our approach is an earlier return to work. Reformat et al. showed an average return to work of 104 days (14.9 weeks) for plate and/or screw fixation of the phalangeal fractures [10]. That is almost three times as long as the results in our patients treated with the intramedullary screw osteosynthesis. This is a significant aspect since these fractures occur mostly in the working population and they can have a negative impact on the patient’s socio-economic wellbeing [11].

A distinct advantage of our technique is sparing the articular surface of the MCP joint. If a screw is introduced from distal through the PIP joint, the surface defect can reach up to 8.5% [8]. Long-term effects of the loss of this articulating surface require further studies.

Only two cases were reported with major extension lag at the proximal interphalangeal joint (PIP) after preforming screw osteosynthesis [7]. On the other hand, re-operations by the plate osteosynthesis reach up to 42% mostly because of adhesions or joint stiffness [12]. Both studies clearly show that the removal of the plate is often necessary.

In our study, the removal of the screw was necessary only in three cases (17.6%) with one patient suffering from a dislocation of the screw into the MCP joint and two patients reporting a disturbing sensation.

On the contrary, plate removal was performed in 13 cases (93%) mostly because of adhesions with corresponding extension lag.

This tendency clearly shows that the removal of the screws is only necessary in exceptional cases whereas plate removal is performed on a routine basis.

We acknowledge that there are some weaknesses in our study. Our cohort is relatively small. Moreover, the study design is not prospective and randomized. Nevertheless, the benefits of the procedure are apparent even for such a small cohort of patients.

While it is acceptable to use unequal sample sizes in Mann–Whitney *U* test, the reliability of the test suffers as the sample sizes become too unequal. In our case, there might be a statistical bias.

## Conclusion

Minimally invasive screw osteosynthesis does not only have the advantage of significantly shorter sick leave, but also shows remarkably improved postoperative range of motion. In contrast to plate osteosynthesis, removal of the screw is only necessary in exceptional cases. With the antegrade approach, even difficult fractures close to the base of phalanges can be treated without destroying the articular surface. Although with plate osteosynthesis a better anatomical and radiological reposition can be acquired, this does not seem to be clinically relevant. We recommend plate osteosynthesis for proximal phalangeal fractures to be considered only in exceptional cases.
